# Protective Effect of Gallotannin-Enriched Extract Isolated from Galla Rhois against CCl_4_-Induced Hepatotoxicity in ICR Mice

**DOI:** 10.3390/nu8030107

**Published:** 2016-02-23

**Authors:** Jun Go, Ji Eun Kim, Eun Kyoung Koh, Sung Hwa Song, Ji Eun Sung, Hyun Ah Lee, Young Hee Lee, Yong Lim, Jin Tae Hong, Dae Youn Hwang

**Affiliations:** 1 Department of Biomaterials Science, College of Natural Resources and Life Science/Life and Industry Convergence Research Institute, Pusan National University, Miryang 627-706, Korea; ydrener@gmail.com (J.G.); prettyjiunx@naver.com (J.E.K.); haha414@naver.com (E.K.K.); rabit357@naver.com (S.H.S.); cnzkcnzk16@hanmail.net (J.E.S.); donc99@hanmail.net (H.A.L.); 2 Department of Organic Material Science and Engineering, Pusan National University, Busan 609-735, Korea; youngheelee@pusan.ac.kr; 3 Department of Clinical Laboratory Science, College of Nursing and Healthcare Science, Dong-Eui University, Busan 614-714, Korea; yonglim@deu.ac.kr; 4 College of Pharmacy and Medical Research Center, Chungbuk National University, Cheongju 361-763, Korea; jinthong@chungbuk.ac.kr

**Keywords:** hepatotoxicity, hepatic fibrosis, Galla Rhois, inflammation, oxidative stress

## Abstract

To investigate the toxicity, protective effects, and action mechanism of gallotannin-enriched extracts isolated from Galla Rhois (GEGR) against carbon tetrachloride (CCl_4_)-induced hepatotoxicity in Institute for Cancer Research (ICR) mice, alterations in serum biochemical indicators, histopathological structure, antioxidative status, hepatic apoptosis-related proteins, and liver fibrosis regulating factors were measured in mice pretreated with GEGR for five days before CCl_4_ injection. The GEGR/CCl_4_ treated group showed decreased levels of three serum marker enzymes (ALP, AST, and ALT) representing liver toxicity, although LDH levels remained constant. Necrotic area indicating hepatic cell death significantly inhibited, while malondialdehyde (MDA) concentration and superoxide dismutase (SOD) expression were dramatically recovered in the GEGR preadministrated group. In mechanism analyses of GEGR, the formation of active caspase-3 and enhancement of Bax/Bcl-2 expression was effectively inhibited in the GEGR/CCl_4_ treated group. The level of pro-inflammatory cytokines, TNF-α and IL-6, as well as the phosphorylation of p38 and JNK in the TNF-α downstream signaling pathway was rapidly recovered in the GEGR/CCl_4_ treated group, while anti-inflammatory cytokine (IL-10) increased slightly in the same group. Furthermore, the GEGR/CCl_4_ treated group showed a significant decrease in collagen accumulation results from alleviation of MMP-2 expression, TGF-β1 secretion and the phosphorylation of Smad2/3. Taken together, these results suggest that GEGR may induce remarkable protective effects against hepatic injury induced by CCl_4_ treatment through upregulation of the anti-inflammatory and antioxidant system.

## 1. Introduction

The liver is the primary organ responsible for metabolism of drugs and chemicals including paracetamol, also known as acetaminophen, diclofenac, and *N*-nitrosocompounds, as well as the primary target of many toxic chemicals such as carbon-tetrachloride (CCl_4_) and aflatoxin [1]. CCl_4_ is a well-known hepatotoxin that induces hepatic damage, necrosis [2], and apoptosis [3]. The administration of CCl_4_ for long periods leads to fibrosis, cirrhosis, and hepatic carcinoma [4]. Furthermore, CCl_4_-induced liver injuries are mediated through the activation of cytochrome P450 to produce reactive intermediates such as trichloromethyl free radical (CCl_3_^•^) and trichloromethyl peroxy radical (CCl_3_OO^•^). These radicals can bind to cellular molecules (nucleic acids, proteins, and lipids), impairing lipid metabolism and initiating hepatic cancer [5]. Therefore, the inhibition of production and activation of free radicals is considered a very important factor for the prevention and treatment of CCl_4_-induced liver injury.

Many natural products have recently received attention as key sources of antioxidants because they are highly active in the prevention and treatment of diseases induced by oxidative stress. However, a wide range of drugs has not been applied to treat or prevent hepatic disorders or investigate their molecular mechanisms until now [6,7,8]. Galla Rhois (GR), which is the excrescence formed by parasitic aphids, primarily *Schlechtendalia chinensi*s Bell, on the leaf of sumac, *Rhus javanica* L. (Anacardiaceae) has been investigated as a natural product with favorable ethno pharmacological properties [9,10]. In Korea, GR has long been used as a traditional medicine for treatment of diarrhea, seminal emissions, excessive sweating, bleeding, and chronic cough, although there is little scientific evidence supporting these pharmacological effects [11,12,13]. Recent studies have revealed the therapeutic effects of GR against various human diseases and their mechanisms. Several compounds and extracts purified from GR exhibited good antibacterial activity against many pathogenic bacteria strains, including *Salmonella* spp., *Escherichia coli*, *Eimeria tenella*, *Brucella abortus*, *Staphylococcus aurous*, and *Clostridium perfringens* [14,15,16,17,18,19]. Methyl gallate and ethyl gallate isolated from GR were also found to exert significant anti-inflammatory activity in lipopolysaccharide (LPS)-stimulated RAW264.7 macrophages via the induction of heme oxygenase 1 and suppression of iNOS/COX-2 [20,21]. Moreover, galloylglucose (GG6-10) isolated from GR inhibited the invasion of metastatic HT-1080 cells into a reconstituted basement membrane via inhibition of gelationolysis mediated by MMP-2 and -9, while the ellagic acid extracted from GR showed anticancer activity against nasopharyngeal carcinoma cells through downregulated expression of COX-2 and stathmin [22,23]. Furthermore, oral administration of 85% methanol extract of GR reduced brain infarct volume by 37.5% and lipid-peroxidation in middle cerebral artery occlusion, while also improving sensory motor function in a transient focal cerebral ischemia rat model [24]. Moreover, tacrine, nitrofurantoinm, and *tert*-butyl hydroperoxide-induced hepatotoxicity in HepG2 cells was greatly retained by two hepatoprotective constituents of GR, an equilibrium mixture of 3-galloyl-gallic acid, 4-galloyl-gallic acid isomers, and 1,2,3,4,6-penta-*O*-galloyl-β-d-glucose [25,26]. However, the protective effects and their mechanisms against hepatotoxicity induced by oxidative stress after toxicant treatment have not yet been investigated in animal models.

Therefore, this study was conducted to investigate the protective effects of Galla Rhois against liver injury induced by CCl_4_ treatment. Gallotannin-enriched extracts isolated from Galla Rhois (GEGR) significantly inhibited the increase in serum biochemical markers of liver toxicity, histopathological damage, apoptosis induction, and hepatic fibrosis through suppression of oxidative stress and upregulation of antioxidant enzymes.

## 2. Experimental Section

### 2.1. Preparation of GEGR

Briefly, samples of GR were collected from plantations in the Hongcheon area of Korea during October of 2013 and dried in a hot-air drying machine (JSR, Seoul, Korea) at 60 °C. Voucher specimens of GR (WPC-14-001) were deposited in the functional materials bank of the PNU-Wellbeing RIS Center at Pusan National University. GEGR was prepared using a modified version of previously described methods [27,28,29,30]. Briefly, dry samples of GR were reduced to powder using an electric blender, after which water extract then obtained at 90 °C for 9 h in a fixed liquor ratio (solid GR powder/water ratio, 1:10) using circulating extraction equipment (IKA Labortechnik, Staufen, Germany). The extracts were subsequently filtered through a 0.4 μm filter, after which they were concentrated by vacuum evaporation and lyophilization using circulating extraction equipment (IKA Labortechnik, Staufen, Germany). Finally, the powder was dissolved in distilled water (dH_2_O) to 1 mg/mL, then further diluted with phosphate buffered saline (PBS) to the required concentration.

### 2.2. Analysis of Gallic Acid, Methyl Gallate, and Gallotanin Concentration

During analysis of the main components of GEGR, gallic acid monohydrate (IUPAC name; 3,4,5-trihydroxybenzoic acid, MW: 170.12 g/mol, Sigma-Aldrich Co., St. Louis, MO, USA), methyl gallate (IUPAC name; methyl 3,4,5-trihydroxybenzoate, MW: 184.15 g/mol, Sigma-Aldrich Co.) and gallotanin (IUPAC name; (3,5-dihydroxy-2-(3,4,5-trihydroxybenzoyl)oxy-6-[(3,4,5-trihydroxybenzoyl)oxymethyl]oxan-4-yl) 3,4,5-trihydroxybenzoate, MW: 1701.20 g/mol, Sigma-Aldrich Co.) were used as standard compounds. The wavelengths of the maximum absorption of pure gallic acid, pure methyl gallate, commercial gallotannin, and gallnut extract were 212/257, 214/268, 213/278, and 212/275 nm, respectively. The UV-VIS spectra of pure gallic acid, pure methyl gallate, pure gallotannin, and the gallnut extract showed two bands at 212–214 nm and 257–278 nm, which were both assigned to the π→π* transitions of the given aromatic units and C=O groups in the UV-VIS region [31]. Finally, the UV-Vis spectra were analyzed using a curve-resolving technique based on linear least-squares analysis to fit the combination of the Lorentzian and Gaussian curve shapes.

The HPLC analysis was conducted as previously described [32]. The HPLC system used for these experiments consisted of a Summit Dual-Gradient HPLC System (Dionex, Sunnyvale, CA, USA) with a PDA UV-vis detector at the Korea Bio-IT Foundry Busan Center. Separation was carried out on an YMC-Triart C18 column (S-5 mm/12 nm, 150 mm × 4.6 mm I.D.) maintained at 40 °C. The mobile phase consisted of solvent A (0.4% formic acid in water) and solvent B (acetonitrile). The gradient condition of the mobile phase was as follows: 0–5 min, 10% B; 5–6 min, 10%–15% B; 6–40 min, 15% B; 40–41 min, 15%–30% B; 41–50 min, 30% B; 50–55 min, 30%–10% B; 55–60 min, 10% B. The injection volume was 5 mL in full loop injection. The flow rate was 0.8 mL/min, and detection was performed at 280 nm.

### 2.3. Design of Animal Experiment

The animal protocol used in this study was reviewed and approved by the Pusan National University-Institutional Animal Care and Use Committee (PNU-IACUC; Approval Number PNU-2014-0662). All mice were handled in the Pusan National University-Laboratory Animal Resources Center, which is accredited by the Korea Food and Drug Administration (FDA), in accordance with the Laboratory Animal Act (Accredited Unit Number-000231) and AAALAC International according to the National Institutes of Health guidelines (Accredited Unit Number; 001525). Ten-week-old male ICR mice were purchased from Samtako (Osan, Korea) and provided with *ad libitum* access to a standard irradiated chow diet (Samtako) consisting of moisture (12.5%), crude protein (25.43%), crude fat (6.06%), crude fiber (3.9%), crude ash (5.31%), calcium (1.14%) and phosphorus (0.99%) and water throughout the feeding study. During the experiment, mice were maintained in a specific pathogen-free state under a strict light cycle (lights on at 08:00 h and off at 20:00 h) at 23 ± 2 °C and 50% ± 10% relative humidity.

ICR mice were divided into the following five groups (*n* = 8 per group): no treated group, vehicle/CCl_4_ treated group, low concentration GEGR (LGEGR)/CCl_4_ treated group, medium concentration GEGR (MGEGR)/CCl_4_ treated group and high concentration GEGR (HGEGR)/CCl_4_ treated group. The first group did not receive any extracts throughout the study period, while the second group repeatedly received a constant volume of desterilized water. The other three groups were treated with 250 mg (LGEGR/CCl_4_ treated group), 500 mg (MGEGR/CCl_4_ treated group), and 1000 mg (HGEGR/CCl_4_ treated group) doses of GEGR per kg of body weight/day. The GEGR was diluted in distilled water and administered via oral gavage for five days. After final administration of GEGR, the No treated group was administered a constant volume of olive oil by intraperitoneal (IP) injection as a control, while the remaining groups were injected IP with a single dose of CCl_4_ (0.7 mL/kg body weight diluted 1:9 in olive oil). At 24 h after CCl_4_ injection, mice in each group were euthanized using a chamber filled with CO_2_ gas, after which blood and organ samples were collected for further analysis.

### 2.4. Serum Biochemistry

After the final administration, all ICR mice in each group were fasted for 8 h, after which blood was collected from the abdominal veins and incubated for 30 min at room temperature. Whole blood was then centrifuged at 1500× *g* for 15 min to obtain the serum, after which serum biochemical components including alkaline phosphatase (ALP), alanine aminotransferase (ALT), aspartate aminotransferase (AST), blood urea nitrogen (BUN), and creatinine (CRE) were assayed using an automatic serum analyzer (Hitachi 747, Tokyo, Japan). All assays employed fresh serum and were conducted in duplicate.

### 2.5. Histological Analysis

Liver tissue was dissected from mice and fixed in 4% neutral buffered formaldehyde (pH 6.8) overnight, after which each liver was dehydrated and embedded in paraffin. Next, a series of liver sections (4 μm) was cut from paraffin-embedded tissue using a Leica microtome (Leica Microsystems, Bannockburn, IL, USA). These sections were then deparaffinized with xylene, rehydrated with ethanol at a graded decreasing concentration of 100%–70%, and finally washed with distilled water. The slides with liver sections were stained with hematoxylin (Sigma-Aldrich Co.) and eosin (Sigma-Aldrich Co.), then washed with dH_2_O, after which the necrotic area was measured using the Leica Application Suite (Leica Microsystems, Wetzlar, Germany).

### 2.6. Immunohistochemical Analysis

Immunohistochemical analysis was performed as previously described [33]. Briefly, liver tissue samples for the detection of collagen proteins using light microscopy were fixed in 4% formaldehyde for 12 h, embedded in paraffin, and sliced into 4 μm thick sections. These sections were then de-paraffinized with xylene, rehydrated, and pretreated for 30 min at room temperature with PBS-blocking buffer containing 10% goat serum. Next, the sections were incubated with anti-collagen antibody diluted 1:1000 in PBS-blocking buffer. The antigen-antibody complexes were then visualized with biotinylated secondary antibody (goat anti-rabbit)-conjugated HRP streptavidin (Histostain-Plus Kit) (Zymed, South San Francisco, CA, USA) at a dilution of 1:1500 in PBS blocking buffer. Finally, collagen proteins were detected using a stable DAB (Invitrogen Corp., Carlsbad, CA, USA) and Imaging Densitometer (Model GS-690, BioRad, Richmond, CA, USA).

### 2.7. Determination of Malondialdehyde (MDA) Levels

The MDA levels were assayed using a Lipid Peroxidation (MDA) Assay Kit (Sigma-Aldrich Co.) according to the manufacturer’s protocols. Briefly, the liver tissue was homogenized in MDA Lysis Buffer containing butylhydroxytoluene (BHT), after which the homogenates were stored at −20 °C until analysis. The sample or standards and TBA solution (70 mM thiobarbituric acid and 5 M glacial acetic acid) were incubated in microcentrifuge tubes at 95 °C for 60 min, then cooled to room temperature in an ice bath for 10 min, after which the reaction absorbance at 532 nm was read using a Vmax plate reader (Molecular Devices, Sunnyvale, CA, USA).

### 2.8. Western Blot

Proteins prepared from the liver tissue of mice were separated by 4%–20% sodium dodecyl sulfate-polyacrylamide gel electrophoresis (SDS-PAGE) for 2 h, after which the resolved proteins were transferred to nitrocellulose membranes for 2 h at 40 V. Each membrane was then incubated separately at 4 °C with the following primary antibodies overnight: anti-Bcl-2 (Abcam, Cambridge, UK), anti-Bax (Abcam), anti-caspase-3 (Cell Signaling, Danvers, MA, USA), anti-MMP-1 (Santa Cruz), anti-MMP-2 (Santa Cruz), anti-MMP-9 (Santa Cruz), anti-Smad 2/3 (Cell Signaling), anti-p-Smad2/3 (Cell Signaling), anti-p38 (Cell Signaling), anti-p-p38 (Cell Signaling), anti-SAPK/JNK (Cell Signaling), anti-p-SAPK/JNK (Cell Signaling), SOD (Abcam), and anti-actin antibody (Sigma-Aldrich Co.). The membranes were subsequently washed with washing buffer (137 mM NaCl, 2.7 mM KCl, 10 mM Na_2_HPO_4_, and 0.05% Tween 20) and incubated with horseradish peroxidase (HRP)-conjugated goat anti-rabbit IgG (Invitrogen Corp.) at a 1:1000 dilution and room temperature for 1 h. Membrane blots were developed using Amersham ECL Select Western Blotting detection reagent (GE Healthcare, Little Chalfont, UK).

### 2.9. Enzyme-Linked Immunosorbent Assay (ELISA) for Tumor Growth Factor (TGF)-β1

The concentration of TGF-β1 in blood serum was measured using a TGF-β1 ELISA kit (Biolegend, San Diego, CA, USA) according to the manufacturer’s protocols. Briefly, the sample for analysis was prepared by adding acidification solution and neutralization solution to liver tissue homogenate progressively. The liver samples or standards and buffer C were incubated in a 96-well plate at room temperature for 2 h while shaking at 200 rpm, after which 100 μL of TGF-β1 detection antibody solution was added to each well and samples were incubated at room temperature for 1 h with shaking. After washing, 100 μL of avidin-HRP D solution was added to each well and the plate was incubated at room temperature for 30 min with shaking. Next, 100 μL of substrate solution was added to each well and the plate was incubated for 10 min in the dark. The reaction was then quenched by the addition of 100 μL of stop solution, after which the plates were analyzed by evaluation of the absorbance at 450 nm using a Vmax plate reader (Molecular Devices, Sunnyvale, CA, USA).

### 2.10. RT-PCR

RT-PCR was conducted to measure the relative quantities of mRNA for inflammatory cytokines including TNF-α, IL-6 and IL-10. Briefly, the liver tissues were chopped with scissors and homogenized in RNAzol solution (Leedo Medical, Houston, TX, USA). The isolated RNA was then measured by UV spectroscopy (BioSpec-Nano) (Shimadzu Scientific Instruments, Columbia, MD, USA). Expression of inflammatory cytokines was assessed by RT-PCR with 3 μg of total RNA from the liver tissue of each group. Next, 500 ng of the oligo-dT primer (Invitrogen Corp.) was annealed at 70 °C for 10 min. Complementary DNA, which was used as the template for further amplification, was synthesized by the addition of dATP, dCTP, dGTP, and dTTP with 200 units of reverse transcriptase. Next, 10 pM each of the sense and antisense primers were added, and the reaction mixture was subjected to 25–30 cycles of amplification in a Perkin-Elmer Thermal Cycler as follows: 30 s at 94 °C, 30 s at 62 °C, and 45 s at 72 °C. RT-PCR was also conducted using GAPDH-specific primers to ensure RNA integrity. The primer sequences for TNF-α expression were as follows: sense, 5′-CCT GTA GCC CAC GTC GTA GC-3′ and antisense, 5′-TTG ACC TCA GCG CTG AGT TG-3′, IL-6: sense, 5′-TTG GGA CTG ATG TTG TTG ACA-3′ and antisense, 5′-TCA TCG CTG TTC ATA CAA TCA GA-3′, IL-10: sense, 5′-CCA AGC CTT ATC GGA AAT GA-3′, and antisense, 5′-TTT TCA CAG GGG AGA AAT CG-3′, GAPDH: sense, 5′-CTC ATG ACC ACA GTC CAT GC-3′, and antisense, 5′-TTC AGC TCT GGG ATG ACC TT-3′. The experiment was repeated three times, and all samples were analyzed in triplicate. The final PCR products were separated by 1.2% agarose gel electrophoresis and visualized by ethidium bromide staining.

### 2.11. Statistical Analysis

One-way ANOVA was used to identify significant differences between the No and CCl_4_ treated groups (SPSS for Windows, Release 10.10, Standard Version, Chicago, IL, USA). In addition, differences in the responses of the vehicle/CCl_4_ treated group and GEGR/CCl_4_ treated groups were evaluated using a *post hoc* test (SPSS for Windows, Release 10.10, Standard Version). All values are reported as the mean ± standard deviation (SD), and a *p*-value of < 0.05 was considered significant.

## 3. Results

### 3.1. Detection of Gallotannin in GEGR

**Figure 1:**
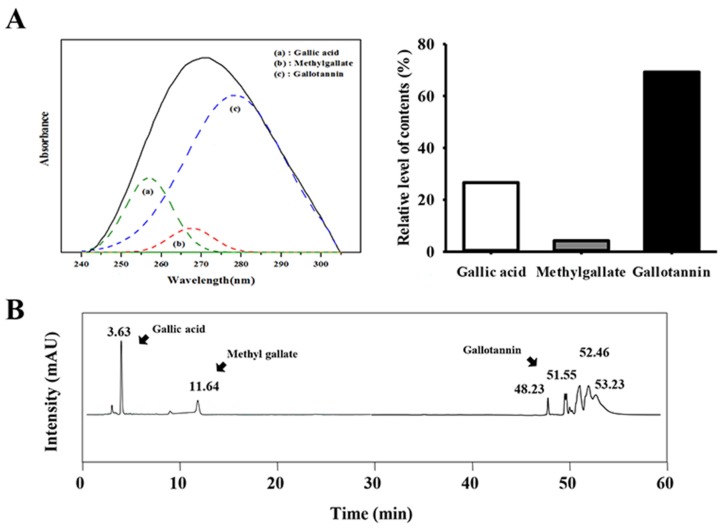
Analysis of major components in GEGR. The contents of gallotannin, gallic acid, and methyl gallate in GEGR were determined based on the (**A**) UV-Vis spectra and (**B**) high-performance liquid chromatography.

Three major compounds, gallic acid, methyl gallate, and gallotannin, were detected in GEGR at different wavelengths (257 nm, 268 nm and 278 nm) by UV-vis spectra. As shown in Figure 1A, gallotannin was present at the highest levels in GEGR (69.2%), followed by gallic acid (26.6%) and methyl gallate (4.2%). GEGR was composed only these three compounds without any significant contamination of other compounds. The chemical formula of gallic acid was very similar to that of methyl gallate, whereas gallotannin formed from the reaction of glucose with dimers or higher oligomers of gallic acid. Also, HPLC curves of GEGR were revealed that there characteristic peaks for gallic acid (3.58 min), methyl gallate (11.4 min), and gallotannin (48.26, 51.55, 52.46, and 53.23 min). These results demonstrate that GEGR contained a high concentration of only three bioactive components that were likely related to its hepatoprotective effects.

### 3.2. Protective Effects of GEGR against Hepatic Toxicity

**Figure 2:**
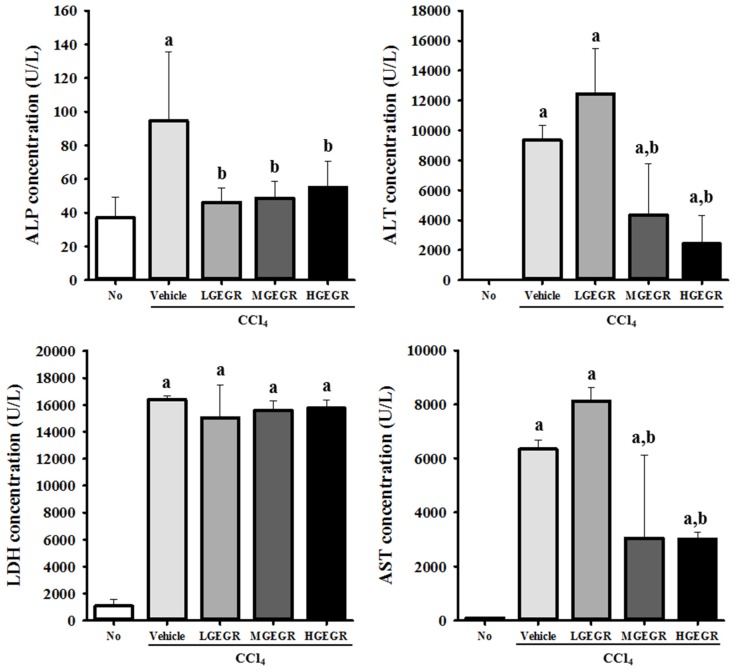
Biochemical markers of hepatic toxicity. Blood was collected from the abdominal veins of vehicle/CCl_4_ and three GEGR/CCl_4_ treated ICR mice (no treatment and HGEGR group (*n* = 7), LGEGR group (*n* = 6), vehicle and MGEGR group (*n* = 8)). Serum concentrations of ALP, AST, ALT, and LDH were analyzed in duplicate using a serum biochemical analyzer as described in the Materials and Methods. Data represent the means ± SD of three replicates. a, *p* < 0.05 relative to the no treatment group; b, *p* < 0.05 relative to the vehicle/CCl_4_ treated group.

To investigate the protective effects of GEGR against CCl_4_-induced toxicity in terms of serum biochemical indicators, the levels of four indicators, ALP, AST, ALT, and LDH, were measured in vehicle/CCl_4_ and GEGR/CCl_4_ treated ICR mice. The levels of ALP, AST, ALT, and LDH were higher in vehicle/CCl_4_ treated rats than no treatment rats. The levels of all factors except LDH were significantly lower with average 54.5% in GEGR/CCl_4_ treated rats than vehicle/CCl_4_ treated rats, although the rate of decrease varied for each factor (Figure 2).

For ALP, the constant ratio of decrease level was detected in three different concentrations of GEGR treated groups. The levels of ALT and AST decreased significantly in the MGEGR and HGEGR treated group, while no significant change was observed in the LGEGR treated group. The LDH level was maintained at a constant value in all GEGR/CCl_4_ treated groups relative to the vehicle/CCl_4_ treated group (Figure 2). Taken together, these results show that pretreatment with GEGR may induce protective effects against CCl_4_-induced liver damage, although the level of protective effects varied according to each factor.

### 3.3. Protective Effects of GEGR against Alteration of Histopathological Structure

**Figure 3:**
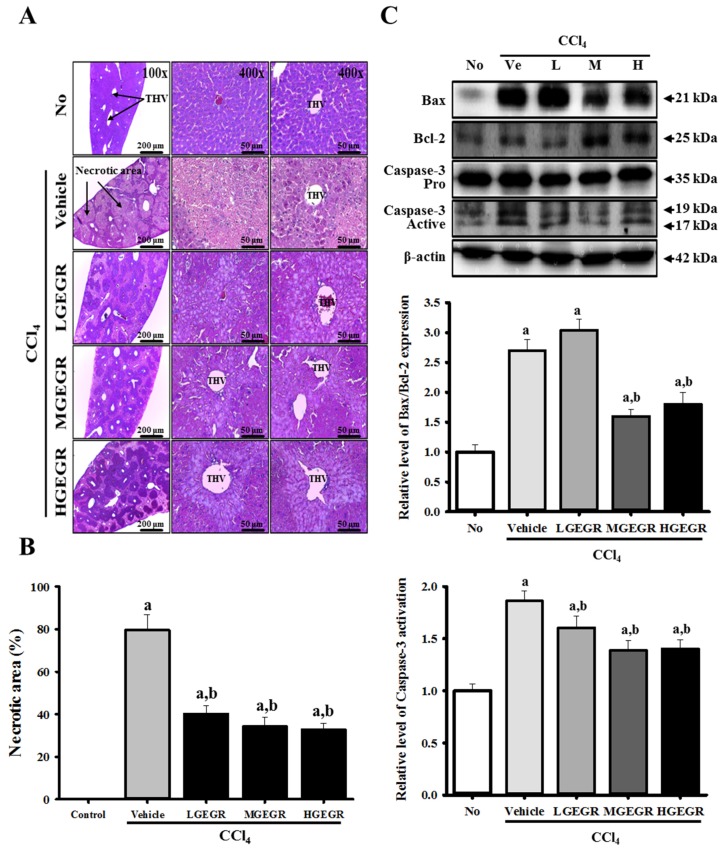
Histology of liver and expression of apoptosis-related proteins. (**A**) Liver tissues collected from ICR mice were fixed in 4% formalin and stained with H&E solution. Liver tissue was mainly observed around the terminal hepatic venule (THV) at a magnification 200× and 400×; (**B**) The necrotic area in each section was observed using Leica Application Suite (Leica Microsystems, Switzerland); (**C**) Expression levels of caspase-3, Bcl-2 and Bax proteins in the liver tissue were analyzed by western blot analysis. Membranes were incubated with antibodies for caspase-3, Bcl-2, and Bax, as well as β-actin protein from the liver. Expression levels were quantified by an imaging densitometer, and the sizes of the products indicated. Data represent the means ± SD of three replicates. a, *p* < 0.05 compared to the no treatment group; b, *p* < 0.05 compared to the vehicle/CCl_4_ treated group.

The differences between tissue components under normal and pathological conditions were visualized by histological staining using hematoxylin and eosin. Histopathological alterations during hepatocellular damage along with the protective effects of GEGR were first identified by histopathological analysis of the liver sections. In the no treatment group, the histopathology of the liver displayed a normal distribution of hepatocytes with clear visible nuclei, a portal triad, and central vein. However, extensive centrolobular necrosis was observed in and around the terminal hepatic venule (THV) of the liver following CCl_4_ treatment. Furthermore, the central vein was significantly dilated in the vehicle/CCl_4_ treated group relative to the no treatment group. However, the liver section of the group pretreated with GEGR for five days displayed low hepatocellular necrosis, a poorly dilated central vein, and regular arrangement of hepatocytes (Figure 3A). Additionally, the necrotic area was dramatically decreased by 51.3%–62.5% in the three GEGR/CCl_4_ treated groups (Figure 3A,B). Thus, our findings indicate that GEGR pretreatment may effectively inhibit the change in histopathological structure of the liver tissue induced by CCl_4_ treatment.

### 3.4. Protective Effects of GEGR on the Regulation of Oxidative Stress

**Figure 4:**
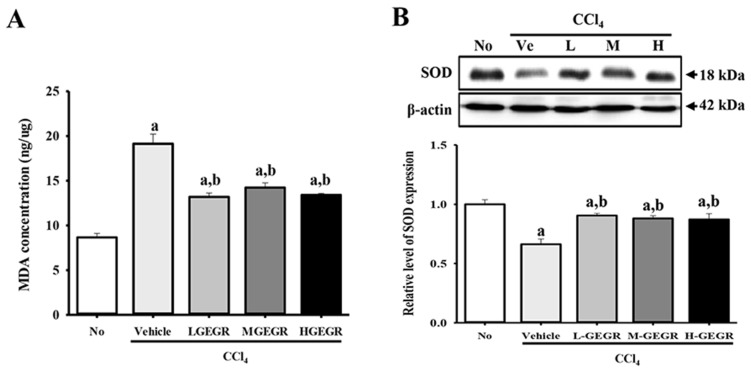
Alteration of oxidant stress related factors. (**A**) The level of MDA was determined in serum collected from mice using a lipid peroxidation assay kit that could detect MDA at 0.1 nmole/mg to 20 nmole/mg; (**B**) The expression level of SOD was measured in the homogenate of liver tissue collected from subset groups. The intensity of each band was determined using an imaging densitometer and the relative level of each protein was calculated based on the intensity of actin protein as an endogenous control. Data represent the means ± SD of three replicates. a, *p* < 0.05 compared to the no treatment group; b, *p* < 0.05 compared to the vehicle/CCl_4_ treated group.

We investigated the protective effects of GEGR on the oxidative stress induced by CCl_4_ exposure. To accomplish this, the MDA concentration and SOD expression were measured in the liver tissue. The concentration of MDA was 221% higher in the vehicle/CCl_4_ treated group than the no treatment group, while the level was significantly lower in the GEGR/CCl_4_ treated groups; however, these levels were not correlated with GEGR dose (Figure 4A). Conversely, an opposite pattern of MDA concentrations was observed upon analysis of SOD expression. The vehicle/CCl_4_ treated group showed a significantly lower level of SOD expression than that of the no treatment group. However, this level was recovered to the level of the no treatment group after GEGR pretreatment (Figure 4B). Taken together, these results indicate that GEGR pretreatment may inhibit the oxidative stress induced by CCl_4_ exposure through the suppression of lipid peroxidation as well as increased SOD repression.

### 3.5. Anti-Apoptosis Effects of GEGR Treatment

To determine if GEGR treatment can prevent the activation of apoptosis induced by CCl_4_ exposure, alterations in the expression of apoptosis-related proteins were examined in the liver tissues of mice that had been pretreated with vehicle or GEGR for five days. Bcl-2 belongs to a family of proteins that includes both pro- and anti-apoptotic members. Among these members, Bcl-2 proteins stimulate anti-apoptosis, while Bax protein significantly inhibits the anti-apoptotic actions of Bcl-2 protein [34,35]. The level of Bax/Bcl-2 expression was higher in the vehicle/CCl_4_ treated group than the no treatment group. However, this level was significantly lower in the MGEGR and HGEGR treated groups than vehicle/CCl_4_-treated group, whereas it was maintained at a constant level in the LGEGR treated group, regardless of GEGR pretreatment (Figure 3C). Conversely, the change in the relative level of active caspase-3 was very similar to that in Bax/Bcl-2. After CCl_4_ injection, the pro-caspase-3 level was reduced, whereas the level of active-caspase-3 increased. In the GEGR/CCl4 treated group, the levels of pro-caspase 3 and active-caspase 3 decreased significantly by 15.1%–27.0% (Figure 3C). Therefore, western blot analysis of apoptotic protein indicated that GEGR pretreatment may protect against hepatocyte apoptosis induced by CCl_4_ injection via regulation of Bax/Bcl-2 expression and caspase-3 activation.

### 3.6. Anti-Inflammation Effects of GEGR Treatment

**Figure 5:**
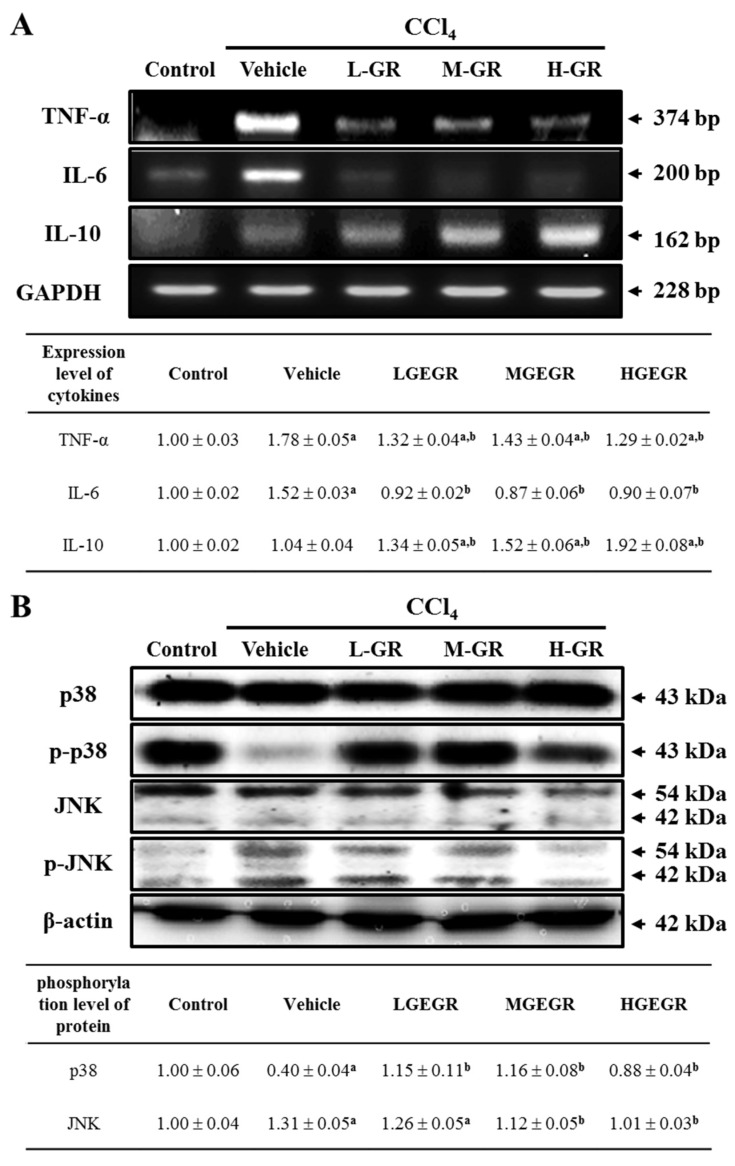
Analysis of inflammation related cytokines and TNF-α downstream signaling pathway. (**A**) Changes in the mRNA level of pro-inflammatory and anti-inflammatory cytokines in the no, vehicle/CCl_4_, and GEGR/CCl_4_ treated group. The mRNA levels of the TNF-α, IL-6 and IL-10 genes were examined by RT-PCR using specific primers; (**B**) Phosphorylation levels of p38 and JNK in liver tissue. To measure the expression levels of each protein, the membranes were incubated with antibodies specific for each protein, as well as β-actin protein from liver lysates. Three mice per group were assayed by western blotting. Values are reported as the means ± SD. a, *p* < 0.05 compared to the No treatment group; b, *p* < 0.05 compared to the vehicle/CCl_4_ treated group.

To investigate the effects of GEGR on inflammation of liver tissue, alterations in the transcript levels of pro- and anti-inflammatory cytokines were confirmed by RT-PCR analysis. Upon analysis of pro-inflammatory cytokines, the level of TNF-α and IL-6 mRNA was higher in the vehicle/CCl_4_ treated group than the no treatment group. However, these levels decreased in the three GEGR treated groups, although the rate of decrease was not dependent on GEGR dose. A complete recovery to that of the no treatment group was only observed for the IL-6 mRNA (Figure 5A). To determine if downregulation of the TNF-α mRNA was accompanied by alterations of the downstream signaling pathway, the phosphorylation levels of the key proteins were measured in the liver tissue. Interestingly, the phosphorylation level of p38 rapidly recovered to that of the no treatment group in the three GEGR treated groups, even though the vehicle/CCl_4_ treated group showed very low levels (Figure 5B). However, the phosphorylation level of JNK was increased in the vehicle/CCl_4_ treated group relative to the no treatment group, and this level decreased in a dose dependent manner in the GEGR treated groups, with those of the MGEGR/CCl_4_ and HGEGR/CCl_4_ treated groups showing complete recovery to that of the no treatment group (Figure 5B). Therefore, these results suggest that the TNF-α signaling pathway activated by GEGR pretreatment was closely related to the regulation of p38 and JNK phosphorylation.

The anti-inflammatory cytokine, IL-10, showed a different pattern from that of the pro-inflammatory cytokines. The level of IL-10 mRNA was not changed in the vehicle/CCl_4_ treated group relative to the no treatment group; however, this level rapidly increased in the GEGR treated groups (Figure 5A). These results suggest that GEGR pretreatment can effectively prevent the inflammatory response induced by CCl_4_ treatment through suppression of pro-inflammatory cytokines and upregulation of anti-inflammatory cytokines.

### 3.7. Protective Effects of GEGR against Liver Fibrosis

**Figure 6:**
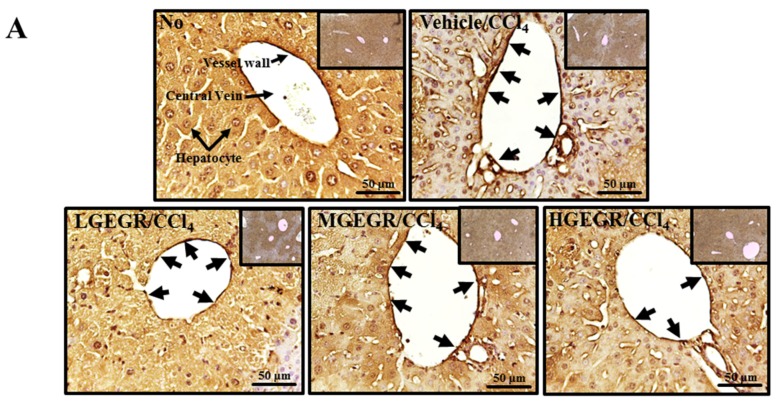

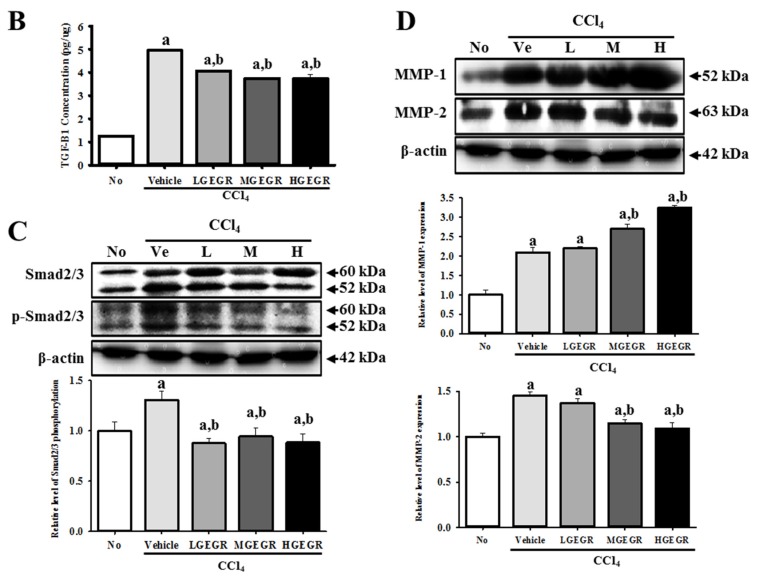
Analysis of the regulatory factors of liver fibrosis. (**A**) Immunohistochemistry of collagen protein. After GEGR/CCl4 pretreatment for 5 days, the liver tissues were collected from all groups and slide sections of liver were prepared as described in the Materials and Methods. The distribution of collagen protein in the slide sections of liver tissue was determined by staining with collagen specific antibody followed by observation at 400×. Black arrows indicate the stained area in THV; (**B**) ELISA for TGF-β1. After collection of blood serum, the concentration of TGF-β1 was determined in the serum collected from the brains of mice using a TGF-β1 ELISA kit that could detect TGF-β1 at 3.5 pg/mL; (**C**) Western blot analysis for Smad2/3 expression. To measure the expression levels of Smad2/3 and p-Smad2/3 protein, the membranes were incubated with specific antibodies for each protein, as well as β-actin protein from liver lysates. Three mice per group were assayed by western blotting; (**D**) Western blot analysis of MMP expression. To measure the expression levels of MMP-1 and MMP-2 protein, the membranes were incubated with specific antibodies for each protein, as well as β-actin protein from liver lysates. Three mice per group were assayed by western blotting. Values are reported as the means ± SD. a, *p* < 0.05 compared to the no treatment group; b, *p* < 0.05 compared to the vehicle/CCl_4_ treated group.

Hepatic fibrosis, which is a histologic hallmark of chronic liver disease, is characterized by the excessive accumulation of connective tissue in the liver and considered an indicator of persistent or progressive hepatic injury [36]. To examine whether GEGR pretreatment can affect collagen accumulation and MMP expression during liver fibrosis induced by CCl_4_ treatment, the levels of collagen and MMP expression were measured in the liver tissue of the subset group. In the case of immunostaining analysis for collagen, the excessive accumulation of collagen became densely stained and expanded in the portal tracts of the vehicle/CCl_4_ treated group when compared with the No treatment group. However, this accumulation was significantly decreased in the three GEGR treated group, even though the highest level of decrease was observed in the HGEGR treated group (Figure 6A). To investigate whether the collagen accumulation can accompany alteration of the collagen regulation enzymes, the MMP 1 and 2 expression was measured in the GEGR/CCl_4_ treated group. The expression level of both proteins was higher in the vehicle/CCl_4_ treated group than the no treatment group; however, the expression level of MMP-1 was significantly higher in the MGEGR/CCl_4_ and HGEGR/CCl_4_ treated group than the vehicle/CCl_4_ treated group, while the LGEGR/CCl_4_ treated group was maintained at a constant level. Upon analysis of MMP-2 expression, a significant decrease was observed in the MGEGR/CCl_4_ and HGEGR/CCl_4_ treated group (Figure 6D). Taken together, these results suggest that GEGR pretreatment may suppress collagen accumulation through downregulation of MMP-2 expression.

The pathogenesis of liver fibrosis is also stimulated by TGF-β1 through promotion of ECM production, which inhibits production of MMPs for effective extracellular matrix deposition in the liver [37]. To investigate the molecular mechanism of the TGF-β1 signaling pathway after GEGR treatment, the concentration of TGF-β1 and the expression level of key proteins in the TGF-β1 signaling pathway were measured in the GEGR/CCl_4_ treated group. The concentration of TGF-β1 in the serum was 392% higher in the vehicle/CCl_4_ treated group than the no treatment group. However, this level decreased dramatically by 18.8%–25.0% in the three GEGRs treated group, even though the concentration of GEGR did not affect this concentration (Figure 6B). Moreover, the treatment of CCl_4_ induced an increase in Samd2/3 phosphorylation level in the TGF-β1 signaling pathway when compared with that of the no treatment group. However, these levels in the three GEGR/CCl_4_ treated group were effectively recovered to that of the no treatment group, even if there was no difference within the three GEGR/CCl_4_ treated groups (Figure 6C). Overall, these results suggest that GEGR pretreatment can reduce the secretion of TGF-β1 and therefore the phosphorylation levels of Smad2/3.

## 4. Discussion

Reactive oxygen species (ROS) are considered an important factor in the pathogenesis of various diseases including atherosclerosis, liver disorders, lung and kidney injury, aging, and diabetes [5]. ROS induced liver disease can be protected and treated by many antioxidants of natural origin, even though their origin varied [38,39]. GEGR has received a great deal of attention as a novel therapeutic drug candidate owing to its strong antioxidant activity. A number of related studies are currently being conducted to identify its novel functions and mechanism of action. In an effort to develop drugs for the treatment of oxidative stress induced diseases, we investigated the protective effects of GEGR in hepatic injury of ICR mice after CCl_4_ exposure. Our results demonstrated that GEGR pretreatment is associated with the prevention of the hepatocytes apoptosis, liver inflammation, and fibrosis via suppression of oxidative stress induced by CCl_4_ treatment.

Until now, composition of GR and their functions were investigated with various studies. Component analysis showed that GR consist of gallic acid, digallic acid, and galloylglucopyranoses (GGs) in ethyl acetate extract [39] and methyl gallate, galloyl-gallic acid isomer, and 1,2,3,4,6-penta-*O*-galloyl-β-d-glucose (PGG) in MeOH extract [25]. Especially, analysis of gallotannin of GR revealed gallotannin were mainly a mixture of mono-tetradeca-GGs, which have antioxidant and antibacterial activity [40,41,42]. Furthermore, PGG, precursor of gallotannin extract from GR showed remarkable hepatoprotective effects than gallic acid, methyl gallate against primary rat hepatocyte necrosis induced by tert-butyl hydroperoxide [26]. PGG also showed significant hepatoprotective effects against tacrine- and nitrofurantoin-induced cytotoxicity in HepG2 cells [25]. Therefore, hepatoprotective effects observed in the present study were considered due to mostly gallotannin rather than gallic acid and methyl gallate.

The present study showed that GEGR pretreatment inhibited serum marker enzymes representing hepatic toxicity in ICR mice with liver injury. Strong decreases in ALT, ALP, and AST in serum were detected in the GEGR/CCl_4_ treated group, although the LDH level was maintained at a constant level (Figure 3). These findings were consistent with those of many previous studies that showed administration of several antioxidant mixtures significantly decreased the levels of serum marker enzymes relative to vehicle groups in CCl_4_ treated animals. For example, the level of ALT, ALP, and AST was significantly decreased by methanol extract of *Carissa opaca* leaves (MCL) [43], aqueous leaf extract of *Persea Americana* (AEPA) [44], *Berchemia lineate* ethanol extract (BELE) [45], methanol extract of *Melaleuca styphelioides* (MSE) [46], and polyphenols-enriched extract from Huangshan Maofeng green tea (HMTP) [47]. However, MCL treatment induced a decrease in LDH level [43], which may have been caused by differences in the composition or concentration of bioactive compounds among studies.

Another conclusion of this study is that GEGR pretreatment applied to the CCl_4_-induced liver injury of ICR mice stimulated a protective effect of hepatocytes against apoptosis and necrosis. Similar results have been reported for the CCl_4_-induced liver injury of ICR mice treated with several bioactive mixtures or compounds. A marked change in liver histopathology and increase in the expression level of apoptotic proteins including caspase-3, Bax, and Bcl-2 were induced by CCl_4_ injection. However, protective effects against CCl_4_-induced acute liver damage were observed in animals treated with water-soluble extract of *Salvia miltiorrhiza* [24], dibenzoyl glycoside from *Salvinia natans* [48], dioscin [49], glycyrrhizic acid [50], and the flavonoid fraction from *Rosa laevigata Michx* fruit [51]. Therefore, the results of present study provide additional evidence that GEGR treatment may induce the attenuation of CCl_4_-induced liver damage.

Oxidative stress induced by CCl_4_ exposure can regulate numerous redox-sensitive transcription factors such as NF-κB, activator protein 1 (AP-1) and early growth response 1 (EGR1). NF-κB activation was tightly associated with the release of pro-inflammatory cytokines including TNF-α, IL-6, and acute phase reactants (C-reactive protein, CRP) [52,53]. CCl_4_ treated animals showed a significant increase in TNF-α and IL-6 concentration; however, these levels were inhibited by several compounds and mixtures including curcumin [54], polysaccharide from *Tarphochlamys affinis* (PTA) [55], and ursolic acid [56]. The results of the present study are in agreement with the above reports, although the rate of decrease varied. However, there are conflicting results regarding the expression of anti-inflammatory cytokines (IL-10) in CCl_4_ injected animals. The IL-10 level in the hepatic supernatant of SD rats with CCl_4_-induced liver injury did not change in response to treatment with ginseng extract and ginsenoside Rb1 for two weeks, although CCl_4_ injection alone induced a slight decrease in their level [57]. However, the IL-10 level in the serum of Balb/c mice with CCl_4_-induced liver injury was significantly reduced by pretreatment with ruxolitinib (15 mg/kg) for 2 h, whereas the levels in the CCl_4_ injected group were higher than in the control group [58]. In this study, the expression of IL-10 was dramatically enhanced by GEGR pretreatment in a dose dependent manner, which differed from the results of previous studies. We thought that this difference in IL-10 expression could be caused by distinction of the properties of bioactive compounds and the genetic background of the experimental animal.

Liver fibrosis is known to be a common response of the liver to a variety of damage including toxic, infectious, or metabolic agents. Fibrosis is also characterized by excessive accumulation of extracellular matrix (ECM) through the abnormal regulation of connective tissue synthesis and ECM homeostasis [59,60]. Many herbs or their bioactive compounds, such as Fufang-Liu-Yue-Qing [61], epigallocatechin-3-gallate (EGCG) [62], silymarin [63] and curcumin [64], have been shown to reduce the severity of hepatic fibrosis in mice and rats. Indeed, the level of TGF-β1, MMP-2 expression and collagen was significantly reduced in response to treatment with most of the above herbs or compounds. Conversely, our finding that GEGR pretreatment induced the effective attenuation of hepatic injury is very similar to the results of previous studies, although the rate of decrease differed.

Lipid peroxidation is considered a critical factor in the pathogenesis of CCl_4_ induced hepatic injury [43]. Therefore, many approaches for the detection of hepatic injury include measurement of lipid peroxides [43]. A significant decrease in lipid peroxides in liver tissues following cotreatment with CCl_4_ and antioxidants indicated the protective effects of many antioxidant compounds and mixtures such as MCL [43], polysaccharide from *Angelica* and *Astragalus* (AAP) [65], ellagitannin-enriched *Melaleuca styphelioides* Sm. (Myrtaceae) [46], and *Fagonia schweinfurthii* (Hadidi) Hadidi (Family: *Zygophyllaceae*) [66] by scavenging of free radicals produced by CCl_4_ injection. In the present study, GEGR pretreatment caused a significant decrease in MDA level, as shown in Figure 4A. These findings are in complete agreement with the results of previous studies and provide novel evidence of the effects of GEGR on lipid metabolism.

SOD decrease the concentration of highly reactive superoxide radicals by transforming them to H_2_O_2_, while catalase (CAT), glutathione (GSH), and glutathione peroxidase (GPx) degrade H_2_O_2_ to protect the liver tissue from ROS produced by CCl4 exposure [43,46]. After decrease of the activity of SOD by CCl_4_ injection, the activity and level of antioxidant enzymes was dramatically recovered in response to treatment with MSE [46], MCL [43], HMTP [47], AAP [65] and *Fangonia schweinfurthii* (Hadidi) Hadidi extract [66]. The present study is the first to demonstrate the alteration of SOD expression in the liver of GEGR/CCl_4_ treated mice. Fundamentally, GEGR pretreatment attenuates the decrease of SOD expression relative to the vehicle/CCl_4_ treated group. These results are concordant with those of previous studies showing the correlation between the level of antioxidant enzymes and the administration of antioxidant mixture.

We investigated the protective effects of GEGR against CCl_4_-induced hepatic injury of ICR mice. GEGR pretreatment prevented the increase of serum marker enzymes, enhancing hepatic apoptosis and necrosis, liver fibrosis, and inflammation through the suppression of lipid peroxidation and induction of antioxidant enzyme expression. Therefore, the results of this study indicate that GEGR should be considered a candidate for the treatment and prevention of hepatotoxicity in the liver.
